# Stress and Strain in Perovskite/Silicon Tandem Solar Cells

**DOI:** 10.1007/s40820-023-01019-3

**Published:** 2023-03-02

**Authors:** Kong Liu, Zhijie Wang, Shengchun Qu, Liming Ding

**Affiliations:** 1 Key Laboratory of Semiconductor Materials Science (CAS), Beijing Key Laboratory of Low Dimensional Semiconductor Materials and Devices, Institute of Semiconductors, Chinese Academy of Sciences, Beijing, 100083 People’s Republic of China; 2 Center of Materials Science and Optoelectronics Engineering, University of Chinese Academy of Sciences, Beijing, 100049 People’s Republic of China; 3 Center for Excellence in Nanoscience (CAS), Key Laboratory of Nanosystem and Hierarchical Fabrication (CAS), National Center for Nanoscience and Technology, Beijing, 100190 People’s Republic of China

Tandem solar cells based on metal halide perovskites are advancing rapidly during last few years [1–17]. The certified power conversion efficiency (PCE) for monolithic perovskite/silicon tandem solar cell reaches 32.5% [18]. Since tandem solar cells contain more layers than single-junction solar cells, stress/strain control is an issue during fabrication and further practical operation. The stress can not only affect the stability of the perovskite layer but also change the optoelectronic properties of the films [19–23].

The mismatch for thermal expansion coefficients or lattice constants induces the stress in stacked films. However, a variety of organic and inorganic materials with different thermal expansion coefficients and lattice constants are needed in perovskite/silicon tandem solar cells. The thermal expansion coefficients for perovskite and organic layers are generally one order of magnitude higher than transparent conductive oxide (TCO) films and glass substrate [19, 24]. Several stimuli can induce compressive or tensile stress in films. For example, residual stress can form during physical deposition and thermal annealing of the perovskite film. Additionally, when the device is applied in a practical environment, the temperature change or external force can form stress in the films [25]. The following equation presents the relationship between stress (*σ*_*ΔΤ*_) and temperature changes (*ΔΤ*) [19]:

1 $$ \sigma_{\Delta T} = \frac{{E_{f} }}{{1 - v_{f} }}\left( {a_{s} - a_{f} } \right)\Delta T $$

where *E*_*f*_ and *ν*_*f*_ are the Young’s modulus and Poisson’s ratio for the films; *α*_*f*_ and *α*_*s*_ are the thermal expansion coefficients for the films and the substrate. The typical *E*_*f*_, *ν*_*f*_ and thermal expansion coefficient of perovskite are 20.5 GPa, 0.33 and 6.1 × 10^–5^ K^−1^, respectively, and the typical thermal expansion coefficient of glass is 3.7 × 10^–6^ K^−1^ [19, 24]. If the temperature decreases from 80 to 30 °C, a tensile stress of 87.66 MPa can form in perovskite film.

**Fig. 1:**
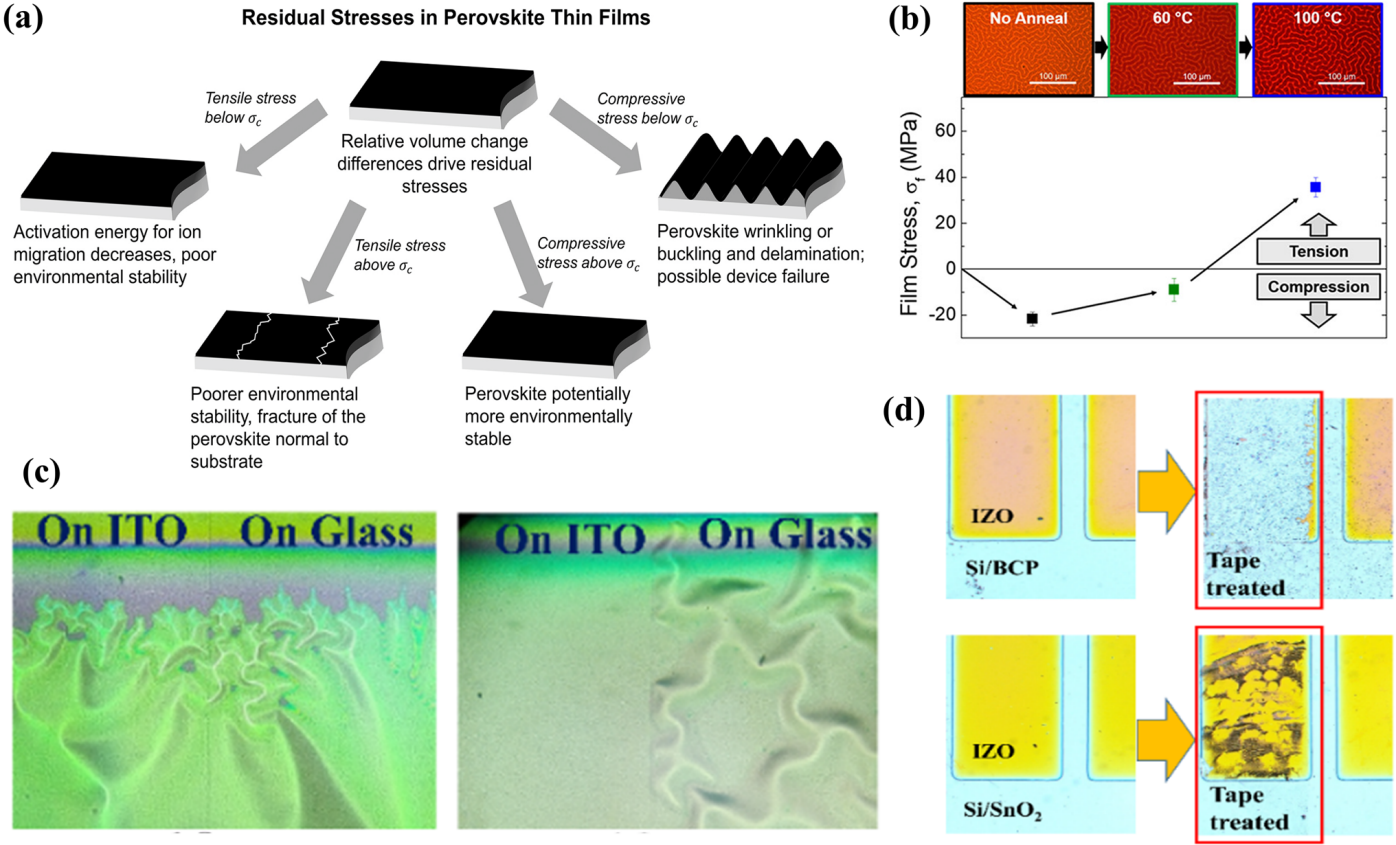
**a** Schematic for stresses and their effects on perovskite films. Reproduced with permission [19], Copyright 2021, American Chemical Society. **b** Wrinkled perovskite film induced by strong compressive stress. Reproduced with permission [26], Copyright 2018, American Chemical Society. **c** Wrinkled IZO film induced by compressive stress transmitted from perovskite layer. **d** IZO films treated by tape, demonstrating that SnO_2_ has better affinity with IZO than BCP. Reproduced with permission [29], Copyright 2022, The Royal Society of Chemistry

Stress in films can cause problems in perovskite/silicon tandem solar cells. If the stress is strong enough, the structure of the tandem device may be destroyed. For the perovskite layer, the compressive stresses greater than the critical stress (*σ*_*c*_) can cause layer failure or delamination (Fig. 1a, b) [19, 26, 27]. Moreover, the strain in the perovskite layer may result in stress in adjacent layers [28]. The sputtered indium zinc oxide (IZO) electrode on perovskite/silicon tandem solar cell surface exhibited peeling-off or wrinkling due to the strong stress induced by the bombardment of sputtered high-energy particles (Fig. 1c) [29]. Notably, the perovskite film coated on glass showed less strain than the perovskite film coated on indium tin oxide (ITO)/glass, which could be attributed to different bonding strengths between the perovskite and substrates due to different roughness of ITO and glass surface [24]. The wrinkled IZO electrode can form under compressive stress induced by strain in the perovskite layer. To mitigate the stresses and overcome film delamination, some methods were adopted, e.g., using a buffer layer with better affinity (Fig. 1d), matching lattice constants and thermal expansion coefficients for the films, or reducing the particle energy and substrate temperature during physical deposition [30]. Encapsulation can help to mitigate stress-induced harm to the devices [31].

**Fig. 2:**
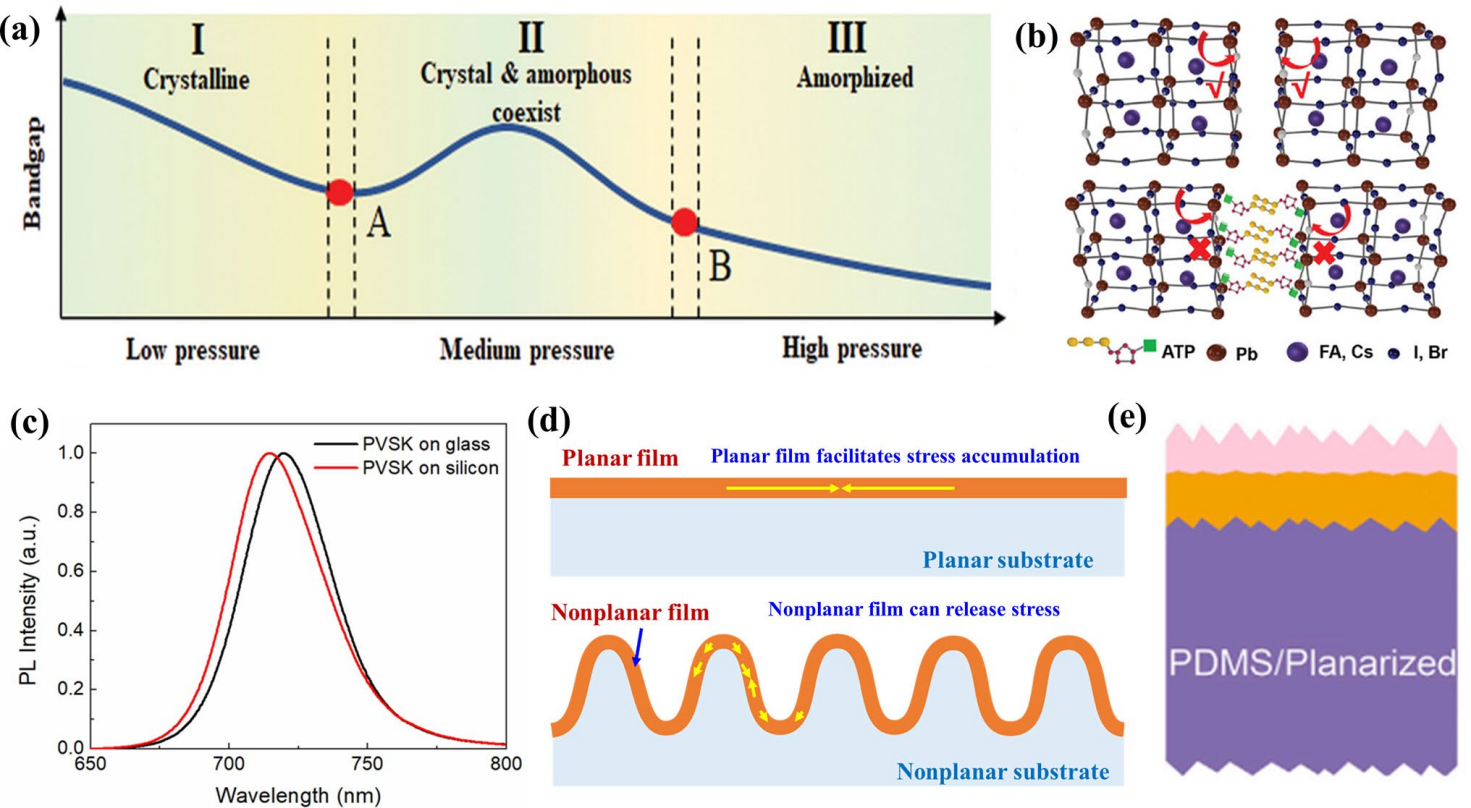
**a** Stress changes the bandgap of perovskites. Reproduced with permission [22], Copyright 2020, Wiley–VCH. **b** Strain reduction in perovskite by introducing ATP. Reproduced with permission [39], Copyright 2022, Wiley–VCH. **c** PL for perovskites on glass and silicon substrates. Reproduced with permission [40], Copyright 2018, Elsevier. **d** Nonplanar structure for stress relaxation in films. **e** Nonplanar silicon top surface can facilitate doctor blading of large-area perovskite films. Reproduced with permission [44], Copyright 2020, Elsevier

Even if stress does not cause peeling-off or wrinkling, it may cause other results. In general, moderate stress can cause dislocations or defects at the interface, leading to enhanced recombination of photogenerated carriers. At the same time, the stress can facilitate light-induced phase segregation of wide-bandgap perovskite or vary the lattice structure of the perovskite directly, leading to bandgap change (Fig. 2a) [22, 31–38]. Wang et al. [39] modulated strain in perovskite/silicon tandem solar cells by adding adenosine triphosphate (ATP) (Fig. 2b). Owing to the enhanced energy barrier for ion migration and reduced light-induced phase segregation in wide-bandgap perovskite, the devices exhibited a remarkable long-term operational stability, keeping 83.60% of the initial PCE after 2500 h. Chen et al. [40] found that the photoluminescence (PL) peak of the perovskite film on silicon exhibited blue shift compared with that on glass substrate (Fig. 2c). Different substrates cause different levels of lattice mismatching between the perovskite and the substrates, leading to different stress and bandgap in perovskite. Because the bandgap of the absorber is a significant factor influencing current matching between sub-cells, such bandgap changes should be noted when investigating single-junction perovskite solar cells on a glass substrate while tandem solar cells on silicon bottom cells.

A possible strategy to reduce the stresses in tandem solar cells is that the films in top cells are formed in a nonplanar structure rather than planar structure. Because stress accumulation can occur more easily in a planar film than in a nonplanar structure. Fortunately, the surface of silicon bottom cells can be textured and served as a light-trapping structure, which provides a foundation for nonplanar structuring. However, most textured structures have sharp edges and corners, which do not facilitate thin film formation. In addition, such structures may lead to the formation of spots with large local stress or defects. Therefore, a nonplanar light-trapping structure without sharp edges and corners may be needed in future perovskite/silicon tandem solar cells [41–43]. As exhibited in Fig. 2d, after introducing such structure, the stress in the films can be relaxed easily even if the device is undergoing external force impact. A textured structure with smoothed edges and corners can realize large-area perovskite film fabrication via doctor blading method (Fig. 2e) [44].

Perovskite itself can form local stress/strain in its lattice. Goldschmidt tolerance factor (*t*), (*R*_A_ + *R*_X_)/[$\sqrt 2$ (*R*_B_ + *R*_X_)], is used to evaluate the crystal structure of perovskites [19, 23], where *R*_A_, *R*_X_, and *R*_B_ are the radius of ions at A, X, and B positions of perovskites (ABX_3_). An ideal cubic structure can be formed when 0.8 < *t* < 1. For certain B and X ions, A cation should have a proper size; otherwise, large strain will be formed in the lattice, resulting in phase transition or instability of perovskite films. For perovskites with APbI_3_ structure, A position can be MA^+^ (CH_3_NH_3_^+^, *t* = 0.83) or FA^+^ ((NH_2_)_2_CH^+^, *t* = 0.99). However, FA^+^ has a larger size than MA^+^, which will lead to a larger size mismatch and local lattice strain. To relax stress in FAPbI_3_ and stabilize the lattice, some small ions, e.g., MA^+^, Rb^+^, or Cs^+^, are incorporated in A position [19, 22, 45–47].

In short, the stress and strain are issues in developing perovskite/silicon tandem solar cells. The issues become severe when the device area is enlarged, causing current mismatch in the sub-cells. Strategies should be proposed to solve these issues.
